# HERNIOPLASTY WITH AND WITHOUT MESH: ANALYSIS OF THE IMMEDIATE COMPLICATIONS IN A RANDOMIZED CONTROLLED CLINICAL TRIAL

**DOI:** 10.1590/S0102-67202015000300002

**Published:** 2015

**Authors:** Mariano PALERMO, Pablo A. ACQUAFRESCA, Miguel BRUNO, Francisco TARSITANO

**Affiliations:** From the Department of General Surgery, Hospital Nacional Profesor Alejandro Posadas, Buenos Aires, Argentina

**Keywords:** Hernia, inguinal, Surgical mesh, Postoperative complications

## Abstract

**Background:**

Inguinal hernia repair is the most common procedure in general surgery and 80,000 operations are performed annually in Great Britain, 100,000 in France and 700,000 in the US. Given its high frequency has a major impact, both in the medical and economic aspects.

**Aim:**

Analyze the immediate postoperative complications comparing mesh versus non mesh hernioplasty.

**Method:**

Randomized control trial, with the enrollment of 263 patients underwent surgery for inguinal hernia randomized by randomization table. Treatment (mesh, Lichtenstein or without mesh, Bassini technique) was assigned using sequentially numbered opaque envelopes having fulfilled the inclusion criteria. The variables analyzed were: postoperative pain, seroma, hematoma, infection, return to normal activities and recurrence.

**Results:**

The mean age was 55.5 years, 88% patients were male and 12% female. The pain was higher in patients operated with mesh.

**Conclusions:**

The inguinal hernia repair mesh group had less immediate postoperative complications and significantly earlier return to work than hernioplasty without mesh, this being one of the most important conclusions.

## Abstract (pt)

**Racional:**

Correção de hérnia inguinal é o procedimento mais comum em cirurgia geral, sendo que 80.000 operações são realizadas anualmente na Grã-Bretanha, 100.000 na França e 700.000 nos EUA. Dada à sua alta frequência tem grande impacto, tanto nos aspectos médicos como nos econômicos.

**Objetivo:**

Analisar as complicações pós-operatórias imediatas comparando hernioplastia com e sem tela.

**Método:**

Ensaio clínico randomizado, com a inclusão de 263 pacientes que foram submetidos à operação de hérnia inguinal, randomizados por tabela de randomização. Os tratamentos foram para o grupo com tela Lichtenstein e ao sem malha técnica de Bassini. Usaram-se envelopes sequencialmente numeradas opacos após terem sido cumpridos os critérios de inclusão. As variáveis ​​analisadas foram: dor pós-operatória, seroma, hematoma, infecção, retorno às atividades normais e recorrência.

**Resultados:**

A idade média foi de 55,5 anos; 88% dos pacientes eram mulheres e 12% homens. A dor foi maior nos pacientes operados com tela.

**Conclusões:**

O grupo com tela teve menos complicações pós-operatórias imediatas e significativamente mais rápido retorno ao trabalho do que hernioplastia sem tela, sendo esta uma das conclusões mais importantes.

## INTRODUCTION

Hernia (Latin, disruption; Greek, bud) it's defined as the organ protrusion through a gap in the abdominal wall. The abdominal wall hernias are the most common cause for major surgery 2 6 10 15 16 20 .

Despite of the high frequency of the surgical repair, surgeons still don't get perfect results and the rate of surgical failure (recurrence) is important and variable 1 3 4 24 . The hernias are among one of the most antique disease that affect men, being one of the first diseases to be detected due to the obvious signs 5 7 10 16 21 . Surgical techniques with mesh or without produce different immediate postoperative complications.

The objective of this study was to analyze the immediate complications of herniplasties with and without mesh, focusing postoperative pain, seroma, hematoma, surgical wound infection and work reinsertion.

## METHODS

The project was reviewed and approved by the Bioethics and Research Committee of the Posadas National Hospital.

This is a randomized controlled clinical trial (RCT) enrolling patients who had indications for hernioplasties with unilateral or bilateral inguinal hernia and met the eligibility criteria before signing the informed consent. They were operated in Surgery Department, Posadas National Hospital, Buenos Aires, Argentina from March 1^st^ 2003 and December 30^th^ 2006.

The inclusion criteria were: nonsurgical risk or liable to compensation; primary inguinal hernia unilateral or bilateral; non complicated hernia; patients without coagulation disorder.

The exclusion criteria were: elevated surgical risk; high blood pressure that not respond to treatment; obstructed hernias; strangulated hernias; recurrent hernias; coagulation disorders

### Surgical randomization

The patients entered to the study in a randomized way and the following techniques were applied: Bassini procedure (without mesh) and Lichtenstein procedure (with mesh). The surgical technique to be performed was contained in sequentially numbered opaque envelopes, using a table of random numbers to produce the series of interventions. The envelopes were in an inviolable dispenser that could only be extracted per unit in a sequentially way, and its extraction was performed before the surgery, when the patient was in the pre-anesthesia room.

All postoperative outpatient monitoring informations were collected in the corresponding tracking forms, which were sent to the external evaluation committee. To evaluate the homogeneity of both study groups the following prognostic variables were taken into account: age, gender, unilateral or bilateral hernia, type of hernia (direct, indirect or mixed), type of work (forced, light) and duration and time of evolution of surgery.

### Sample size 

It was considered to obtain a 50% reduction of immediate complications in experimental group (technique with mesh).

Considering a type I or alpha error of 5%, a Beta or type II error of 20% and a ratio of 1: 1 to conform the two study groups, the total number of patients in each group was estimated at 220. At the end of the fourth year a preliminary analysis of the results was performed by a Monitoring and Data analysis independent committee to re-evaluate the sample size necessary and decided that it wasn't necessary to continue with the inclusion of patients.

### Statistical analysis

To evaluate the quantitative variables (age, disease evolution, duration of surgery) in both groups arithmetic means and standard deviations were calculated, and were compared by the Student's t-test. For the remaining prognostic variables, percentages were calculated which were compared by using the Chi-squared distribution or the Fisher's exact test according to the obtained frequencies. For the ordinal variables, the Chi-squared distribution of lineal association was applied. The relative risk (RR) and confidence intervals of 95% were used for the comparative analysis of the groups operated with mesh versus without mesh. Furthermore, the ARR (absolute risk reduction) and the number needed to treat (NNT) were calculated where corresponded. A survival analysis considering the time to return to work (in days) as the dependent variable of the type of surgery performed (stratification variable) was applied. Median of these times were calculated according to the Kaplan Meier method and compared using the test of equality between strata: Log Rank Test. To perform the adjust for other intervening variables Cox's proportional hazards model was applied, the hazard ratios were calculated and their significance was tested by Likelihood Partial Test. The adequacy of the adjustment was measured by the R 2 coefficient. The data analysis was done by intention to treatment, retaining subjects of study in their respective groups assigned at randomization. The statistical package "STATA" was used to obtain the sample size and performing statistical analyzes.

## RESULTS

Were studied 263 patients undergoing inguinal hernia repair. In 135 cases (51,3%) technique with mesh was applied and in 128 (48,7%) without mesh. After an intermediate analysis the sample size was recalculated and the power was significant not to continue including patients because of the benefits of the mesh placement.

The patient's general characteristics are shown in the Table 1 , in which can be seen that no significant differences were found in the distribution by gender, age and type of work.

**TABLE 1:** Patient's general characteristics according to the type of surgery

Variables	Surgery		p
	With mesh	Without mesh
Age (mean±DS)	55.5 (±16.1)	54.4 (±17.0)	0.59*
Gender (n, %)
Male	119 (88.2)	106 (82.8)	0.22^#^
Female	16 (11.8)	22 (17.2)	
Work type (n, %)
Heavy	75 (55.6)	64 (50)	0.37^#^
Light	60 (44.4)	64 (50)

*t-Student test; ^#^ Chi-square test

Patients in which mesh was not applied showed a nearly fivefold greater risk of developing wound infection when compared to those that underwent with mesh technique surgery (ARR=5.5% NNT=18.1). Every 18 patients operated with the technique with mesh, one wound infection will be prevent ( Table 2 )

**TABLE 2:** Patient's postoperative characteristics according to the type of surgery

Variables	Group		p
	With mesh	Without mesh
Work reinsertion (mean ± DS)	42 (28.2)	106.9 (51.6)	<0.0001*
Wound infection (n, %)
Yes	2 (1.5)	9 (7)	0.031**
No	133 (98.5)	119 (93)	
Recurrence (n, %)
Yes	1 (0.7)	3 (2.3)	0.36**
No	134 (99.3)	125 (97.7)

*t-Student test for unequal variances; ^**^Fishers exact probability test; wound infection QX: relative risk=4.75 (1.05 <RR< 21.55)

**FIGURE 1:**
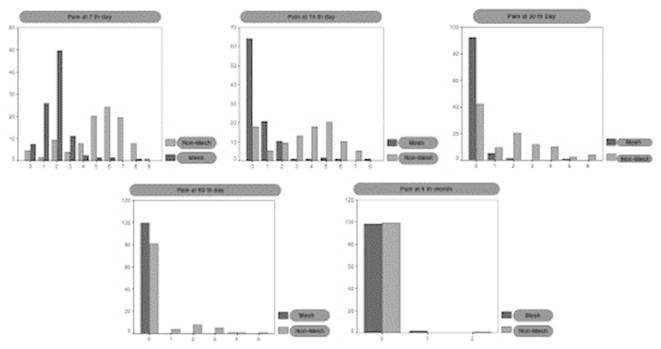
Distribution of patients according to pain intensity and type of surgery

The Figure 1 shows the intensity of pain, measured using the E.V.A. test. As can be seen, greater proportions of patients operated without mesh have high values in this scale.

As for variable pain is considered 0 as no pain, 1 to 3 as mild pain, 4 to 7 as moderate and 8 to 10 as severe. As is can be seen, the proportions of patients with moderate or severe pain were higher in the intervention group without mesh. Association between type of surgery and pain at the 7^th^ day postoperatively was found (Chi-square test: p<0.0001). Mild pain was related to the surgical technique with mesh (7.4% had no pain and 86.7% had mild pain) and moderated pain was related to the technique without mesh (71.9% moderate pain and 8.8% severe pain - Table 3 )

Association between the type of surgery and the pain at the 15^th^ postoperative day was found (Fisher test: p<0.0001). In the group of patients operated with mesh 64.4% had no pain and 31.9% had mild pain, while in the group without mesh only 18% had no pain and 53.9% expressed moderate pain.

Statistically significant association was found between the type of operation and use of analgesics at seven days (Chi-square test: p<0.0001), being this item superior in the patients operated without mesh (87.5 % relative risk=1.60 (1.35<RR < 1.89).

**TABLE 3:** Need for analgesia by type of surgery

Variable	With mesh n %	Without mesh n %	p
Analgesia at the 7th day
Yes	74 54.8	112 87.5	< 0.0001*
No	61 45.2	16 12.5	
Analgesia at the 15th day
Yes	2 1.5	3 2.3	0.68**
No	133 98.5	125 97.7	
Analgesia at month#
Yes	1 0.8	1 0.8	1.00**
No	132 99.2	127 99.2

* Chi-square Test; **Fishers Test; ^#^two missing data

When analyzing the presence of seromas at different evolution time it could be seen that the presence of them did not differ between both groups of patients ( Table 4 ).

**TABLE 4:** Presence of seromas by type of surgery

With mesh n %	Without mesh n %	p
Seroma at 7th day	0.71*
Yes		10 7.4	8 6.2
No		125 2.6	120 93.8
Seroma at 15th day	0.49**
Yes		3 2.2	5 3.9
No		132 97.8	123 96.1
Seroma at month	0.61**
Yes		1 0.7	2 1.6
No	134 99.3	126 98.4

* Chi-square test; **Fishers test

The same happened with the presence of hematomas.

### Survival analysis

**FIGURE 2:**
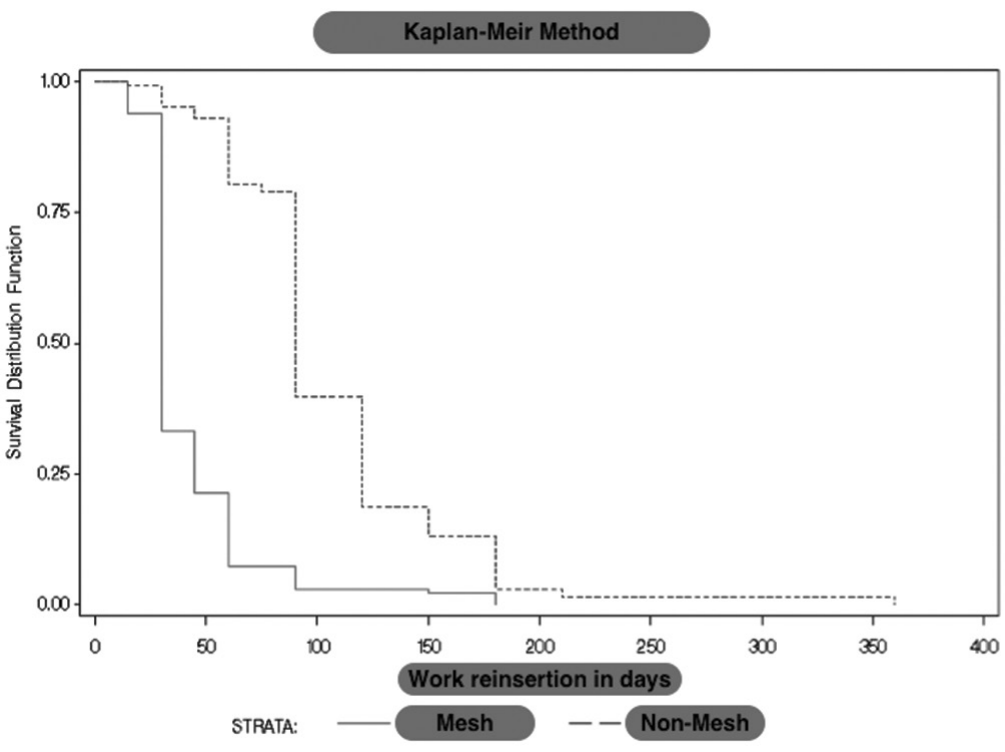
Kaplan-Meier survival function

In Figure 2 , the survival functions for each type of operation are shown (with mesh or without mesh) considering as the dependent variable the time to return to work (in days) and as the event, the work reinsertion. These functions were estimated using Kaplan-Meier proposed methodology. It should be noted that no data were presented censored because all patients had the event.

Time until work reinsertion (in days); event è work reinsertion; strata è type of surgery (with or without mesh).

Significant differences were observed in time to return to work when patients were classified according to the type of surgery (with mesh or without mesh), showing a faster recovery in patients undergoing with mesh technique surgery (Log-rank test: p<0.0001). The median time to return to work (in days) by type of surgery was: with mesh, 30 days versus without mesh 90 days (p=<0.0001). Significant differences were observed in time to return to work when patients were classified according to the type of operation. Patients operated with mesh had an earlier return to work.

## DISCUSSION

When talking about inguinal hernias, choosing a surgical procedure becomes difficult not only by the large number of existing procedures but also because none of them shows an indisputable superiority over the others 2 24 .

In order to choose the technique, the surgeon should be guided by some basic principles, which are: the hernia is a benign disease; with essentially a functional impact and that the operation should not expose the patient to serious complications or sequelae.

The selection of the technique it's done by following three basic criteria 5 : 1) the patient: the tissues solidity and the tension to which the tissues are submitted; 2) the hernia: a small indirect hernia with good muscular wall is very different from a major collapse of the groin with multiple recurrences; 3) the surgeon: the surgical training, experience and degree of specialization.

Inguinal hernia repair is the most common surgery in general surgery 19 , about 80,000 interventions per year are performed in Britain 8 , 100,000 in France 17 and 700,000 in USA 8 . Given its high frequency, inguinal hernia has an important impact on both medical and economic fields 22 .

The standard method for inguinal hernia repair, proposed by Bassini in 1887, has had little change in the last hundred years. The annual statistics from various countries show a recurrence rate of 10-15%, including the most used techniques without mesh as Shouldice, Mc Vay among others^122^.

The concept of tension free hernioplasty, postulated by Lichtenstein, is widely used nowadays. This method, which uses a synthetic mesh, seems to have more beneficial effects than the techniques without meshes, because it's an easier technique, it has less postoperative pain, a faster work reinsertion and it can be performed with local anesthesia.

Among the hernioplasties without mesh, nowadays the Shouldice technique is considered the gold standard, due to its minor percentage of recurrence when compared with other techniques. Even though, most studies have a high percentage of patients lost, demonstrated in a meta-analysis that the best technique within hernioplasty with mesh is the Shouldice, with recurrence rate of 5% 12 18 22 23 .

The choice for the use of prosthetic mesh or not depends largely on the patient's age and the type of hernia. Direct or mixed hernias carry a higher risk of recurrence due to the weakness of the tissue, justifying the placement of the mesh.

The mesh placement rate increased from 7% in 1992 to 51% in 1996 in Sweden 5 9 11 . Nowadays, there is difficulty in choosing a surgical technique for the treatment of inguinal hernia 10 12 22 .

According to the meta-analysis published by Scott N.W. at the Cochrane Library which included 12 randomized controlled clinical trials and whose objective was to compare all surgical techniques with mesh versus techniques without mesh, resulted in a significant reduction of recurrences O.R. 0.39 IC 95% (0.25,0.59). This páper shows that hernioplasty with mesh has approximately 40 times less chance of recurrence (IC 25 to 60 times) compared with techniques without mesh 13 14 20 22 . This meta-analysis has an appropriate methodology, and it is strong scientific evidence that can validate the choice of techniques with mesh if is taken into account the benefit of reducing by 40 times the recurrence in the mesh group versus the group without mesh.

In this study it is clear that the recurrence was significantly lower in patients operated with mesh; so, this variable was not studied in this paper, but it is unclear, due to inconclusive results, the immediate complications such as postoperative pain, seroma, hematoma, wound infection and work reinsertion. The author himself after analysis of the results suggests that could be clarified by conducting an RCT with a larger sample size.

To answer these questions mentioned above (immediate postoperative complications) was performed this research, with the objective of achieving the necessary power to provide adequate scientific response.

## CONCLUSIONS

The hernia repair with mesh due to be a tension-free technique is associated with less pain; patients undergoing surgery without mesh required higher doses of painkillers; seroma and hematoma presence did not differ between both groups; there is a tendency that patients with mesh hernioplasty have a lower rate of infection; and time from surgery to work reinsertion in patients operated with mesh, was shorter, and this is one of the most important conclusions of this paper.
